# Functional prediction of differentially expressed lncRNAs in HSV-1 infected human foreskin fibroblasts

**DOI:** 10.1186/s12985-016-0592-5

**Published:** 2016-08-05

**Authors:** Benxia Hu, Yongxia Huo, Guijun Chen, Liping Yang, Dongdong Wu, Jumin Zhou

**Affiliations:** 1 Key Laboratory of Animal Models and Human Disease Mechanisms of the Chinese Academy of Sciences & Yunnan Province, Kunming Institute of Zoology, Kunming, Yunnan 650223 China; 2 Kunming College of Life Science, University of Chinese Academy of Sciences, Kunming, Yunnan 650204 China; 3 State Key Laboratory of Genetic Resources and Evolution, Kunming Institute of Zoology, Chinese Academy of Sciences, Kunming, Yunnan 650223 China

**Keywords:** RNA-seq, Long noncoding RNA, HSV-1, In cis, In trans

## Abstract

**Background:**

One of the most important functions of long noncoding RNAs (lncRNAs) is to control protein coding gene transcription by acting locally in cis, or remotely in trans. Herpes Simplex Virus type I (HSV-1) latently infects over 80 % of the population, its reactivation from latency usually results in productive infections in human epithelial cells, and is responsible for the common cold sores and genital Herpes. HSV-1 productive infection leads to profound changes in the host cells, including the host transcriptome. However, how genome wide lncRNAs expressions are affected by the infection and how lncRNAs expression relates to protein coding gene expression have not been analyzed.

**Methods:**

We analyzed differentially expressed lncRNAs and their potential targets from RNA-seq data in HSV-1 infected human foreskin fibroblast (HFF) cells. Based on correlations of expression patterns of differentially expressed protein-coding genes and lncRNAs, we predicted that these lncRNAs may regulate, either in cis or in trans, the expression of many cellular protein-coding genes.

**Results:**

Here we analyzed HSV-1 infection induced, differentially expressed lncRNAs and predicted their target genes. We detected 208 annotated and 206 novel differentially expressed lncRNAs. Gene Ontology and Pathway enrichment analyses revealed potential lncRNA targets, including genes in chromatin assembly, genes in neuronal development and neurodegenerative diseases and genes in the immune response, such as Toll-like receptor signaling and RIG-I-like receptor signaling pathways.

**Conclusions:**

We found that differentially expressed lncRNAs may regulate the expression of many cellular protein-coding genes involved in pathways from native immunity to neuronal development, thus revealing important roles of lncRNAs in the regulation of host transcriptional programs in HSV-1 infected human cells.

**Electronic supplementary material:**

The online version of this article (doi:10.1186/s12985-016-0592-5) contains supplementary material, which is available to authorized users.

## Background

Approximately 70 % of the human genome is transcribed, but less than 2 % of which encodes proteins. The remainder, collectively referred to as noncoding RNAs, are one of the most intensely investigated subjects in almost all areas of biomedical science. Based on the size of their transcripts, noncoding RNAs (ncRNAs) are classified into small noncoding RNAs (length <200 nt) and long noncoding RNAs (lncRNAs, length > 200 nt). With their number approaching 10 thousand in the human genome [1, 2], lncRNAs have been shown to have diverse activities, ranging from recruiting chromatin remodeling complexes to transcriptional regulation and post transcriptional processing of RNAs [3]. LncRNAs could cooperate with DNA, other RNAs or proteins [4] to function in cell differentiation, development to diseases [5]. One of the best understood lncRNAs functioning in development, HOTAIR, is located within the HoxC gene cluster on chromosome 12, and represses the expression of genes in the HoxD gene cluster on chromosome 2 [6]. The most heavily investigated imprinted loci, IGF2/H19 encode a lncRNA H19, and its depletion caused precocious muscle differentiation [7]. lnc-DC, on the other hand, is a lncRNA implicated in immune system development, its knockdown impaired Dendritic Cell (DC) differentiation and reduced capacity of DCs to stimulate T cell activation [8]. LncRNA is also implicated in tumorigenesis, for example, a lncRNA ceruloplasmin (NRCP) was highly up-regulated in ovarian tumors, which significantly increased cancer cell growth by altering glycolysis compared with normal cells [9]. MALAT1 (metastasis associated lung adenocarcinoma transcript 1), one of the most abundant lncRNAs, could regulate alternative splicing by modulating the phosphorylation of the serine/arginine splicing factors [10]. LncRNA is also implicated in pathogen-host interaction [11], for example, a cellular lncRNA, negative regulator of antiviral response (NRAV), promoted influenza A virus (IAV) replication and virulence.

An increasing amount of evidence suggests that lncRNAs act either locally to regulate nearby genes in cis or remotely, i.e. over one mega bases away or on a different chromosome, in trans to control the transcription of target genes [2, 4, 12, 13]. LncRNAs could directly silence or activate gene expression, or by indirectly regulate chromatin states of their target genes [14]. For instance, Evf, a cis-acting lncRNA, is required for the activation of Distal-less homeobox (Dlx) 5 and 6 genes and generation of GABAergic interneurons in vivo [15]. HOTAIR, on the other hand, binds to PRC2 and LSD1 complexes and couples H3K27 methylation and H3K4 demethylation activity to hundreds of sites genome-wide [16].

Herpes Simplex Virus type I (HSV-1) is a double strand DNA virus, with a 152 kb genome, encoding about 80 genes, which also include several small RNAs and a lncRNA, latency associated transcript, or LAT [17, 18]. HSV-1 reactivation can cause diseases from the mild cold sores to the crippling encephalitis [19]. During productive HSV-1 infection, which is responsible for these diseases, host cells activate native antiviral immunity [20, 21], apoptosis [22, 23], DNA damage response and other stress responses [24–27] to limit HSV-1 infection and growth [27]. However, many viral genes are designed to modify these responses. For example, ICP4, ICP22 and ICP27 are negative regulators of the host apoptotic response, ICP34.5 inhibits the type I interferon response by inactivating protein kinase R (PKR) [28–30], while ICP8 inhibits the host DNA damage response by inactivating the ataxia-telangiectasia and Rad3 related (ATR) kinase [31–34].

Although the molecular details of the HSV-1 lytic infection process is well understood, many important questions on virus-host interactions and host responses, especially at the transcriptomic level still remain unanswered. Here we analyzed RNA-seq data [35] of HSV-1 infected human HFF cells for differentially expressed lncRNAs, and found 208 annotated and 206 novel lncRNAs in HSV-1 infected cells. Using method described by Derrien et al. [2], we found protein-coding genes (PCGs) that are either negatively or positively correlated with lncRNAs expression. These correlated lncRNAs exist both in cis and in trans relative to their target PCGs, and are mostly involved in chromatin assembly and metabolic process GO terms. Pathway analysis showed that PCGs correlated with lncRNAs in trans were enriched in B cell receptor signaling pathway, Toll-like receptor signaling pathway and RIG-I-like receptor signaling pathway. Pathway analysis of PCGs associated with lncRNAs in cis included axon guidance, focal adhesion, adherens junction and Neurotrophin signaling pathways. Interestingly, genes in two neurodegenerative diseases, Huntington’s disease (HD) and Parkinson’s disease (PD) are potentially regulated by lncRNAs, such as PPP3CB-AS1, SNHG8 and DARS-AS1. This analyses suggested important roles of lncRNAs in HSV-1 infection, and their regulation of cellular gene transcription.

## Methods

### Prediction of lncRNAs

We used FastQC software to filter low quality reads with default argument.

Clean RNA-seq data were aligned to Human reference genome (Homo_sapiens. GRCh38) with Tophat2. Cufflinks and Cuffcompare were used to assemble and compare transcripts with reference. Then we used to custome script to extract the length of transcript ≥ 200 nucleotides, the number of exon ≥ 2 and belonged to class code “i”, “j”, “o”, ”u” and “x”. We used CNCI software to predict the coding capacity of candidate lncRNAs. Based on Sun et al. [36], we obtained high quality assemblies and used it as final reference annotation file. We counted differentially expressed genes with Cuffdiff2, and used the following criterion to select differentially expressed lncRNA genes: FDR ≤ 0.05 and fold-change ≥2 (Additional file 1: Figure S1).

### Targets of lncRNAs

For gene regulation by lncRNA in cis, we extracted PCGs located within a genomic window of 1 Mb as targets of lncRNAs. For in trans regulation of PCGs by lncRNA, we extracted PCGs far away with lncRNA about 1 Mb or located in different chromosomes. Then we calculated the correlation coefficient (r, Pearson, *p* value ≤ 0.05) between the targets and lncRNAs and selected |r| ≥ 0.8 and |r| ≥ 0.9 for cis and trans, respectively (Additional file 1: Figure S1).

### Gene ontology and pathway analysis

We uploaded the targets of lncRNAs into The Database for Annotation, Visualization and Integrated Discovery (DAVID) v6.7 to do Gene Ontology analyses (biological processes) and Pathway analyses (KEGG pathways). DAVID calculated a *p value* for gene enrichment with a modified Fisher’s exact test, and a Benjamin-Hochberg multiple test correction. We selected significant GO terms and pathways with *p value* ≤ 0.05.

### Co-expressed modules analysis

We used STEM software to analyze co-expressed modules of PCGs and lncRNAs, and we chose significant modules with *p value* ≤ 0.05.

### Statistic analysis

We used *R* relative packages, such as pheatmap (pheatmap: Pretty Heatmaps, Raivo Kolde, 2015) and VennDiagram (VennDiagram: Generate High-Resolution Venn and Euler Plots, Hanbo Chen, 2015), and functions, such as cor.test to analyze data and draw figures.

## Results

### LncRNAs expressed in HSV-1 infected HFF cells

To determine how lncRNA expression is affected by HSV-1 infection, we analyzed RNA-seq datasets from NCBI GEO and filtered the low quality reads using FASTX-Toolkit (http://hannonlab.cshl.edu/fastx_toolkit/) software, followed by aligning RNA-seq data onto human reference genome with Tophat2 [37], and subsequently using Cufflinks [38] and Cuffcompare [38] to assemble and compare transcripts. In addition, we extracted the fasta sequences of candidate novel lncRNAs, and used CNCI [39] software to distinguish noncoding RNAs from coding RNAs. Finally, compared to Ensembl database, we obtained 14,654 annotated lncRNAs and 3,050 novel lncRNAs consisted of 5,909 transcripts (Additional file 1: Figure S1).

**Fig. 1:**
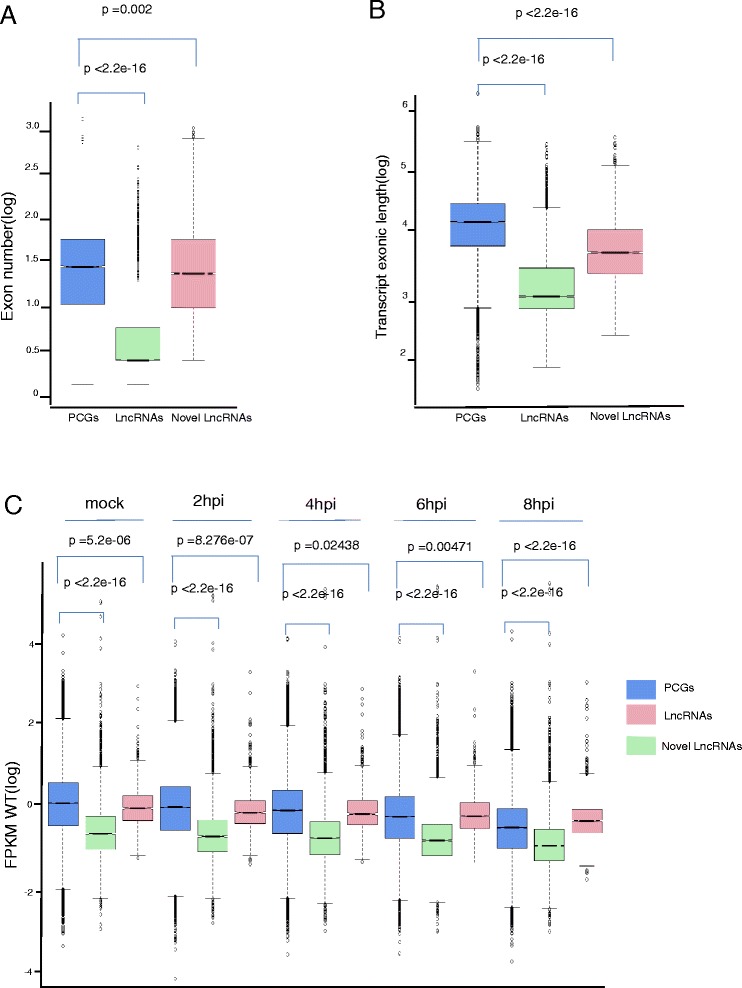
Comparisons of transcript length, exon number and expression levels of HSV-1 infected transcriptome. **a** Comparison of exon number. The novel lncRNAs represent smaller the number of exon than PCGs on average. **b** Comparison of transcript length. The novel lncRNAs show shorter length on average than PCGs. **c** The coding transcripts represent slightly higher expression level than the novel lncRNAs, but significantly higher expression than the annotated lncRNAs. Wilcox.test, *p* value <0.05

Previous studies in mammals showed that lncRNAs are shorter in length, have fewer exons and expressed at much lower levels than PCGs [1, 36, 40, 41]. To determine whether the novel lncRNAs we detected have the same features, we calculated exon numbers in detected novel lncRNAs and PCGs, and found that the exon numbers of these novel lncRNAs are much smaller than that of PCGs (*p* value < 0.05, Welch Two Sample t-test) (Fig. 1a), and the exon length of novel lncRNAs is much shorter than that of PCGs (*p* value < 0.05, Welch Two Sample t-test) (Fig. 1b). We next calculated the expression level of PCGs and novel lncRNAs, and found that the expression level of PCGs is much higher than that of novel lncRNAs in all samples with the exception of 6hpi and 8hpi samples (*p* value < 0.05, Welch Two Sample t-test, Fig. 1c). The expression levels of PCGs from the 6hpi and 8hpi samples were slightly higher than that of novel lncRNAs, which is likely due to the viral protein VHS, which is known to degrade host mRNAs [42–46] (*p* value <0.05, Welch Two Sample t-test). Thus, these novel, de novo assembled lncRNAs are highly credible. We then used the high quality GTF file as reference annotation file for further analysis.

### Differentially expressed lncRNAs due to HSV-1 infection

Cuffdiff2 [47] software was used to calculate differentially expressed (DE) genes from 2hpi to 8hpi samples compared to mock infected samples.

**Fig. 2:**
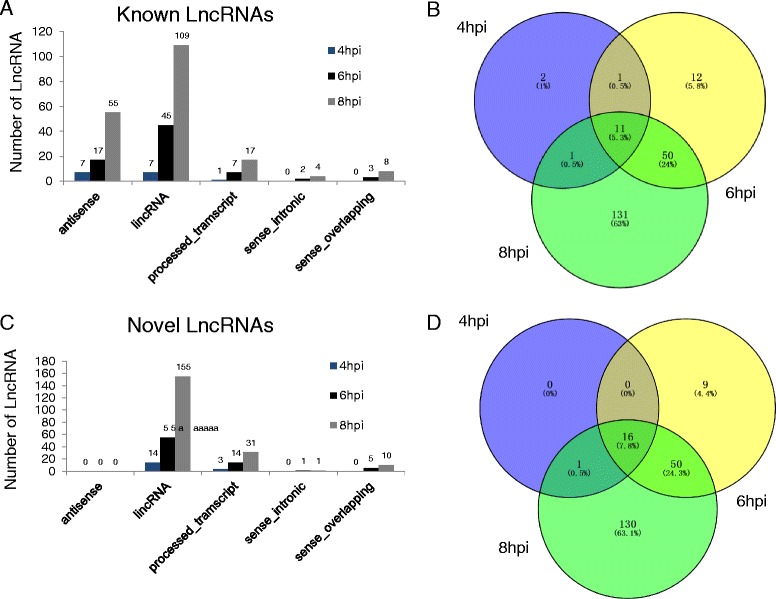
The number of differentially expressed lncRNAs at different time points after HSV-1 infection. **a** There are 15, 74 and 193 annotated differentially expressed lncRNAs in 4hpi, 6hpi and 8hpi samples, respectively. **b** There are 11 common annotated lncRNAs among 4hpi, 6hpi and 8hpi samples. **c** There are 17, 75 and 197 differentially expressed novel lncRNAs in 4hpi, 6hpi and 8hpi samples, respectively. **d** 16 common novel lncRNAs are found among 4hpi, 6hpi and 8hpi samples

Overall, the number of DE lncRNAs increased with time after virus infection. For example, there was no DE lncRNAs at 2 hours after HSV-1 infection, but there were 15 annotated and 17 novel DE lncRNAs expressed at 4hpi sample (Fig. 2a and b). At 6hpi sample, 74 annotated and 75 novel DE lncRNAs were found, respectively (Fig. 2a and b), while in the 8hpi sample, many more DE lncRNAs, including 193 annotated and 197 novel lncRNAs were observed (Fig. 2a and b). We pooled all DE lncRNAs from 4hpi to 8hpi samples, and obtained 414 DE lncRNAs, including 208 annotated and 206 novel lncRNAs (Fig. 2a and b).

Based on methods by Derrien et al. [2], we also classified the above DE lncRNAs into five types: antisense RNA, long intronic non coding RNA (lincRNA), processed transcript, sense intronic transcript, and sense overlapping transcript. We found that the number of lincRNAs was most abundant among all lncRNAs. Previous study showed the expression of lncRNAs is very cell type and tissues specific [2], we therefore determined the temporal specificity of lncRNAs during the course of infection. Indeed, these lncRNA expression patterns are highly dynamic, for example, there were only 27 overlapped DE lncRNAs, including 11 annotated and 16 novel lncRNAs, when compared the 4hpi with 8hpi samples (Fig. 2a and d). This dynamic nature is consistent with their regulatory roles.

### Prediction of cis target genes of virus induced DE lncRNAs

**Fig. 3:**
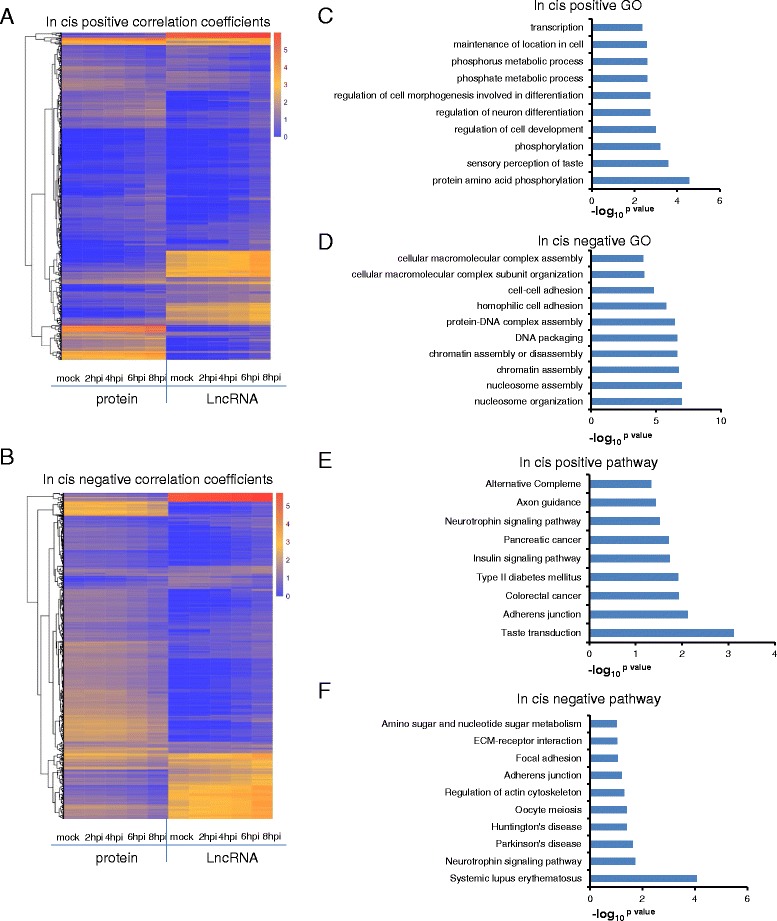
The predicted function of lncRNAs regulating PCGs in cis. **a** 714 PCGs and 83 lncRNAs composed 918 pairs of positive in cis,, the color-axis of heatmap is the log_10_^FPKM+1^. The correlation coefficients (r) ≥0.8, *p* value ≤ 0.05. **b** 933 and 84 lncRNAs composed 1188 negative regulatory modes in cis. The correlation coefficients (*r*) ≤ −0.8, *p* value ≤ 0.05. **c**, **e**. GO analysis and pathway analysis of coding genes associated with positive correlation coefficients, *p* value ≤ 0.05; (**d**, **f**). GO analysis and pathway analysis of PCGs associated with negative correlation coefficients, *p* value ≤ 0.05. The x-axis of c/d/e/f graphs is the –log_10_^*p* value^

Based on previous studies describing in cis and in trans regulation modes to predict target PCGs and functions of lncRNAs at transcriptomic level [2, 48], we analyzed in cis correlation coefficient (r) of expression of lncRNAs and PCGs. Between PCGs and lncRNAs, we obtained 928 and 1,188 pairs of positive and negative regulatory modes, respectively (Fig. 3a and b).

Using DAVID [49] software, Gene Ontology (GO) analysis of DE PCGs with positive cis correlation coefficients with lncRNAs revealed genes enrichment for metabolic processes and neuronal differentiation GO terms (Fig. 3c). In contrast, PCGs negatively correlated with lncRNAs were enriched in assembly of marco molecular complexes, such as nucleosome and chromatin assembly. For example, two PCGs, HIST1H2AB and HIST1H2AC, which were both significantly down-regulated about 2.5 fold after HSV-1 infection, were negatively associated with one annotated lncRNA, ZNRD1-AS1 (significantly up-regulated about 4 fold by the infection) (Fig. 3d).

Pathway (KEGG) enrichment analysis revealed 9 pathways for DE PCGs positively affected by lncRNAs in cis, and 10 pathways for DE PCGs negatively affected by lncRNAs in cis (Fig. 3e and f), which include axon guidance, focal adhesion, adherens junction and Neurotrophin signaling pathways. These pathways are involved in differentiation and survival of neural cells, and higher neuronal function, such as learning and memory [50, 51]. As HSV-1 is a neurotropic virus, the regulation of these genes in cis by HSV-1 induced lncRNAs is of biological significance. One of the immediate early viral protein encoded by HSV-1, ICP0 is known to activate neuronal genes [25, 52–54], and may contribute to the DE lncRNAs and correlated PCGs. Interestingly, we also found PCGs with negative correlation coefficients were enriched in Systemic lupus erythematous, which is reported to be linked to HSV-1 infection [55].

To confirm the co-expression signature of PCGs and lncRNAs by correlation analyses, we used STEM [56] software to analyze co-expressed modules from the above-mentioned expression of PCGs and lncRNAs. In both positive and negative regulatory modules, we obtain 5 and 4 specific profiles showed significant enrichment for PCGs and lncRNAs (*p* value < 0.05), respectively (Additional file 1: Figure S2A and D). GO and Pathway enrichment analysis for these PCGs from significant co-expressed modes (Additional file 1: Figure S2A and C, E and F) showed that the top10 GO terms and pathways were also enriched in chromatin assembly, metabolic processes and neuronal differentiation, and Neurotrophin signaling and adherens junction, respectively. Thus, these results are consistent with the results obtained using correlation analysis.

### Prediction of trans target genes of HSV-1 induced DE lncRNAs

**Fig. 4:**
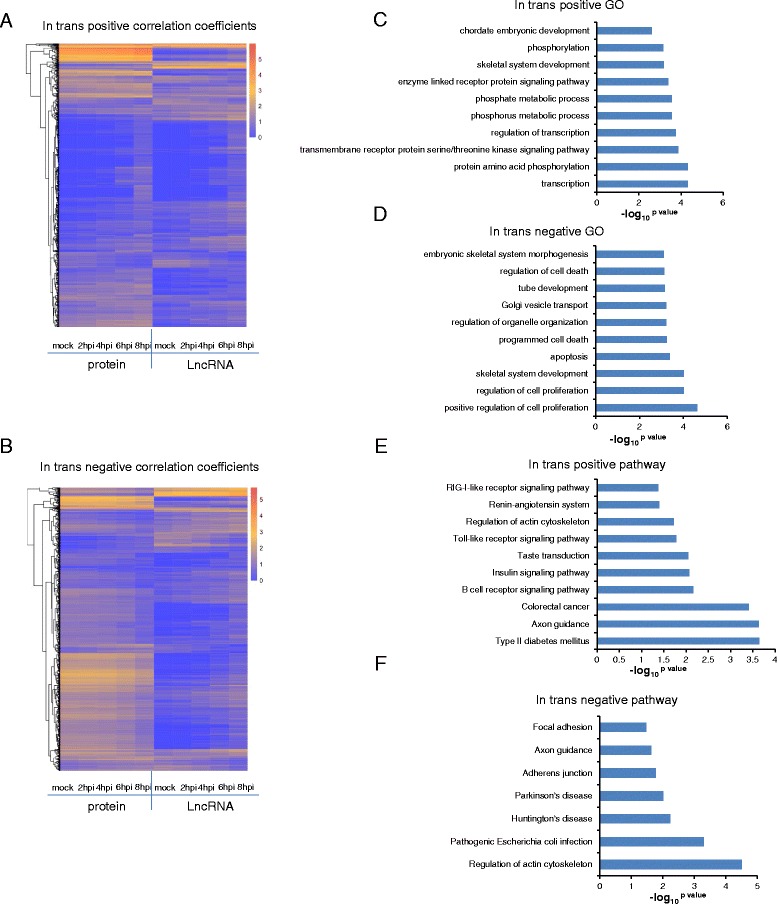
The predicted function of lncRNAs regulating PCGs in trans. **a** 1029 PCGs and 387 lncRNAs composed 2072 pairs of positive in trans, the color-axis of heatmap is the log_10_^FPKM+1^. The correlation coefficients (r) ≥0.9, *p* value ≤ 0.05. **b** 1047 and 369 lncRNAs composed 1803 pairs of negative regulatory modes in trans. The correlation coefficients (*r*) ≤ −0.9, *p* value ≤ 0.05. **c**-**d** GO analysis and pathway analysis of coding genes associated with negative correlation coefficients, *p* value ≤ 0.05; (**e**-**f**). GO analysis and pathway analysis of PCGs associated with positive correlation coefficients, *p* value ≤ 0.05. The x-axis of c/d/e/f graphs is the –log_10_^*p* value^

We then analyzed in trans correlations of expression (defined as pairs consisting of lncRNAs and coding genes separated by a distance of >1 mega base, or located on different chromosomes), and found 2,072 and 1,803 pairs of positive and negative regulatory modes, respectively (Fig. 4a and b).

GO analysis of genes with positive correlation coefficients enriched these genes in transcription, metabolic processes, and development on the top 10 terms (Fig. 4c). For example, one PCG, KDM5B, a lysine-specific histone demethylase from the jumonji/ARID domain-containing family with a role in the transcriptional repression [57], was up-regulated after HSV-1 infection and showed highly positive correlation (*r* > 0.9, *p* value < 0.01) with one annotated lncRNA, KCNQ1OT1. KCNQ1OT1 interacts with chromatin and regulates transcription of multiple target genes through epigenetic modifications [58]. On the other hand, the coding gene, PRKAA1, a member of Ser/Thr protein kinase family [59], positively associated with one novel lncRNA (location of the novel lncRNA in genome: Chr22: 38705722–38794198), which was up-regulated after HSV-1 infection. Again from the imprinted IGF2/H19 loci, IGF2 [60] was up-regulated by HSV-1 infection, and showed highly positive correlation with one antisense lncRNA, BZRAP1-AS1. These positive correlations strongly suggest regulatory connections between lncRNAs and correlated PCGs in chromatin and transcriptional regulation.

Interestingly, we also found genes positively affected by lncRNAs through in trans were enriched in B cell receptor signaling pathway, which resulted in the expression of immediate early genes that further activated the expression of other genes involved in B cell proliferation, differentiation and Ig production as well as other processes [61]. For example, B-cell linker, BLNK, encodes a cytoplasmic linker or adaptor protein that plays a critical role in B cell development [62], was induced after HSV-1 infected HFF cells for 6 h. It exhibited highly positive correlation (*r* > 0.99, *p* value < 0.001) with two novel lncRNAs (located at Chr12:220425–262873 and Chr9:99297947–99319599). Another important enriched pathway is the Toll-like receptor signaling pathway [63]. For example, IRF3, which was significantly down-regulated by HSV-1 infection, associated with one novel lncRNA (located at Chr7: 34,928,699-35,038,271), while IRF5 and IRF7, significantly up-regulated after HSV-1 infected for 8 h, were positively associated with two annotated lncRNAs, ZNRD1-AS1 and MAMDC2-AS1, respectively. A third immunity related pathway is the RIG-I-like receptor signaling pathway, which is responsible for detecting viral pathogens and generating innate immune responses [64, 65] (Fig. 4e). Besides immune genes, we also found lncRNAs functioning in the DNA damage response, for example, protein coding gene ATG5, which was significantly down-regulated after HSV-1 infection, showed positive association with one lncRNA, noncoding RNA activated by DNA damage (NORAD), which is also down-regulated after HSV-1 infection [11]. As HSV-1 infection is known to activate the cellular DNA damage response, the down regulation of NORAD could be due to the active inhibition of one viral gene, ICP8, which inhibits the host DNA damage response by inactivating the ATR kinase [31–34].

In contrast, genes with negative correlation coefficients with lncRNAs were enriched in apoptosis and cell proliferation by GO analysis (Fig. 4d), suggesting that genes in these two processes are likely subject to inhibition by virus induced lncRNAs. For example, CASP7, a member of caspases functioning in apoptosis [66], was down-regulated by HSV-1 infection, and showed negative correlation with three lncRNAs, PPP3CB-AS1, SNHG8 and DARS-AS1. TGFBR2, a member of the Ser/Thr protein kinase family and the TGFB receptor subfamily [67], was down-regulated after HSV-1 infection and showed highly negative correlation (*r* < −0.9, *p* value < 0.01) with KCNQ1OT1, which can interact with chromatin and regulate transcription of multiple target genes through epigenetic modifications [50].

Notably, two neurodegenerative diseases, Huntington’s disease (HD) and Parkinson’s disease (PD) pathway genes are negatively correlated with lncRNAs both in cis and in trans (Fig. 3e and Fig. 4f). HD, a neurodegenerative genetic disorder, can affect muscle coordination and lead to mental decline and behavioral symptoms [68]. Previous studies found that REST (RE1-Silencing Transcription Factor) is involved in HD and is considered a hub in the co-ordinate regulation of the transcriptome and epigenome in HD [69, 70]. Normally, REST represses neuronal genes in non-neuronal tissues [71], but in HSV-1 infected HFF cells, it was significantly down-regulated, leading to up-regulation of many neuronal genes, which is believed to be caused by HSV-1 protein ICP0 [25, 52–54]. PD is another important neurodegenerative disorder [72]. Here UCHL1 (Ubiquitin Carboxyl-Terminal Esterase L1), a susceptibility gene for PD and a potential target for disease-modifying therapies [73], was significantly down-regulated by HSV-1 infection, and a novel, HSV-1 induced lncRNA (located at 4:68282460–68285959), negatively correlated with REST and UCHL1, thus could potentially inhibit the expression of REST and UCHL1. Therefore, HSV-1 induced lncRNAs could promote neural specific programs.

We also analyzed co-expressed modules of PCGs and lncRNA to check whether the correlation analysis is right, we found 8 and 6 significant co-expressed modules for positive and negative regulation (*p* value < 0.05), respectively (Additional file 1: Figure S3A, D). Compared to above mentioned GO terms, this method found that PCGs, co-expressed with lncRNAs, were also enriched in the similar GO terms, such as metabolic processes and development. In addition, pathway enrichment analysis revealed that many PCGs were also enriched in the pathways detected by correlation analysis. For instance, we found that some PCGs, such as BLNK, REST and UCHL1, were enriched in B cell receptor signaling pathway, HD and PD, respectively (Additional file 1: Figure S3B and C, E and F).

## Discussion and conclusions

HSV-1 is an important and ubiquitous human pathogen. Its lytic infection in cultured cells has been used as a paradigm to investigate the basic mechanism of transcription, molecular virology and virus-host interactions. HSV-1 infection is known to profoundly alter the host transcriptome, from differential gene expression, to RNA splicing and RNA Pol II read through [29, 35, 74]. The human genome encodes around ten thousand lncRNAs [16, 17], which are involved in many different biological processes [3, 5]. However, how lncRNA expression is affected by HSV-1 infection and whether lncRNAs play any roles in the transcriptomic response to viral infection are not understood. Here we analyzed the HSV-1 infected transcriptome to reveal how many lncRNAs are expressed in HFF cells and how they are affected by the infection, and found 14,654 annotated lncRNAs, and 3,050 novel lncRNAs when compared to the Ensemble database. Among the 3,050 newly discovered lncRNAs, 789 were induced by HSV-1 infection. Then based on criterion (FDR ≤ 0.05 and fold-change ≥2) of differential expression analysis, we obtained 208 annotated DE lncRNAs, including 166 up-regulated and 42 down-regulated lncRNAs, and 206 novel DE lncRNAs, including 171 up-regulated and 35 down-regulated lncRNAs, after HSV-1 infection. Similar method of predicting novel lncRNAs was used by Sun et al. [36], in order to avoid genome contamination in RNA-seq samples, here we set more strict cutoff that the number of exon of candidate novel lncRNAs is more than 1. As a result, our prediction may have excluded some positive genes.

Next, we predicted the potential targets of the DE lncRNAs by correlation of expression levels of PCGs and lncRNAs. An important modus operandi of lncRNAs is to act in cis to control the expression of PCGs that are positioned in the vicinity of their transcription sites [5]. By correlation prediction of expression levels in cis mode, we found many PCGs negatively associated with lncRNAs were enriched in large marco molecular complex assembly, including protein DNA complex assembly, nucleosome assembly and chromatin assembly, suggesting that these lncRNAs may negatively regulate these PCGs. In contrast, GO analysis showed that PCGs positively correlated with lncRNAs were enriched in metabolic process, differentiation and phosphorylation. These patterns of correlations suggest that these lncRNAs might regulate the correlated PCGs in these biological processes.

HSV-1 is well known to modulate host responses from native immunity [29, 75] to apoptosis [17, 18] to benefit viral transcription and replication. Pathway analysis showed that some PCGs positively related with lncRNAs in trans were enriched in B cell receptor signaling pathway, Toll-like receptor signaling pathway and RIG-I-like receptor signaling pathway. For instance, IRF5 and IRF7, positively correlated with ZNRD1-AS1 and MAMDC2-AS1, may be up-regulated by two lncRNAs, respectively. This analysis suggests that lncRNA might play important roles in regulating immune response. In the list of genes that are predicted to be in trans targets of lncRNAs, we found that PCGs negatively associated with lncRNAs were enriched in apoptosis. For example, PIM2, reported to promote cell survival and inhibit apoptosis [76], was up-regulated by HSV-1 in HFF cells and was negatively correlated with lncRNA DNM3OS. Meanwhile, CASP7, a member of the apoptotic pathway [61], was down-regulated by HSV-1 infection and showed negative correlation with PPP3CB-AS1, SNHG8 and DARS-AS1. Thus, this analysis brings forward a hypothesis that HSV-1 might modulate the host apoptotic pathway by targeting lncRNAs, including DNM3OS, PPP3CB-AS1, SNHG8 and DARS-AS1.

The DNA damage response is another important cellular response to HSV-1 infection. Here ATG5, which functions as a potent molecular decoy for PUMILIO proteins repress a program of genes necessary to maintain genomic stability [11], was significantly down regulated after HSV-1 infected in HFF cells. This expression pattern showed highly positive association with NORAD [10]. HSV-1 infection induces DNA replication stress and activates the DNA damage response [24–26]. At the same time, viral protein ICP8 inhibits the ATR kinase activity to modulate the cellular DNA damage response [31–34]. Thus it would be interesting to investigate whether HSV-1 actively inhibits the expression of NORAD, for example by ICP8 to modulate host response.

In summary, we predicted potential target genes of HSV-1 induced, differentially expressed lncRNAs in HFF cells, and revealed a large number of target genes that may participate in cellular pathway functions in response to viral infection. Our analysis suggests that one lncRNA might regulate many PCGs, while individual PCGs could also be regulated by multiple lncRNAs. The two methods we used, correlation analysis and co-expression module analysis by and large produced similar results, thus these potential lncRNAs target genes offer important clues to further study mechanism of viral-host interaction and the regulatory functions of lncRNAs.

## Abbreviations

DE, differentially expressed; GO, gene ontology; HD, huntington’s disease; HFF, human foreskin fibroblast; HSV-1, herpes simplex virus type i; lncRNA, long noncoding RNA; PCGs, protein-coding genes; PD, parkinson’s disease

## Additional file

Additional file 1: Figure S1. Computational prediction of lncRNAs in HSV-1 infected HFF cells. The computational strategy of predicting of function of lncRNAs in HSV-1 infected HFF cells. Figure S2 The predicted function of lncRNAs based on co-expressed modules in cis. a. there were 5 significant co-expressed modules in positive regulatory model, *p* value ≤ 0.05. b-c. GO analysis and pathway analysis of PCGss co-expressed with lncRNAs, *p* value ≤ 0.05; d. there were 4 significant co-expressed modules in negative regulatory model, *p* value ≤ 0.05. e-f. GO analysis and pathway analysis of PCGs co-expressed with lncRNAs, *p* value ≤ 0.05. Figure S3 The predicted function of lncRNAs based on co-expressed modules in trans. a. there were 8 significant co-expressed modules in positive regulatory model, *p* value ≤ 0.05. b-c. GO analysis and pathway analysis of PCGss co-expressed with lncRNAs, *p* value ≤ 0.05; d. there were 6 significant co-expressed modules in negative regulatory model, *p* value ≤ 0.05. e-f. GO analysis and pathway analysis of PCGs co-expressed with lncRNAs, *p* value ≤ 0.05. (DOCX 653 kb)
