# Supplementary material for: Functional prediction of differentially expressed lncRNAs in HSV-1 infected human foreskin fibroblasts
Source: Virol J. 2016 Aug 5;13:137. doi: 10.1186/s12985-016-0592-5 (PMC4974703; doi:10.1186/s12985-016-0592-5)
Supplement: Additional file 1: Figure S1. — Computational prediction of lncRNAs in HSV-1 infected HFF cells. The computational strategy of predicting of function of lncRNAs in HSV-1 infected HFF cells. Figure S2 The predicted function of lncRNAs based on co-expressed modules in cis. a. there were 5 significant co-expressed modules in positive regulatory model, p value ≤ 0.05. b-c. GO analysis and pathway analysis of PCGss co-expressed with lncRNAs, p value ≤ 0.05; d. there were 4 significant co-expressed modules in negative regulatory model, p value ≤ 0.05. e-f. GO analysis and pathway analysis of PCGs co-expressed with lncRNAs, p value ≤ 0.05. Figure S3 The predicted function of lncRNAs based on co-expressed modules in trans. a. there were 8 significant co-expressed modules in positive regulatory model, p value ≤ 0.05. b-c. GO analysis and pathway analysis of PCGss co-expressed with lncRNAs, p value ≤ 0.05; d. there were 6 significant co-expressed modules in negative regulatory model, p value ≤ 0.05. e-f. GO analysis and pathway analysis of PCGs co-expressed with lncRNAs, p value ≤ 0.05. (DOCX 653 kb) [file 12985_2016_592_MOESM1_ESM.docx]

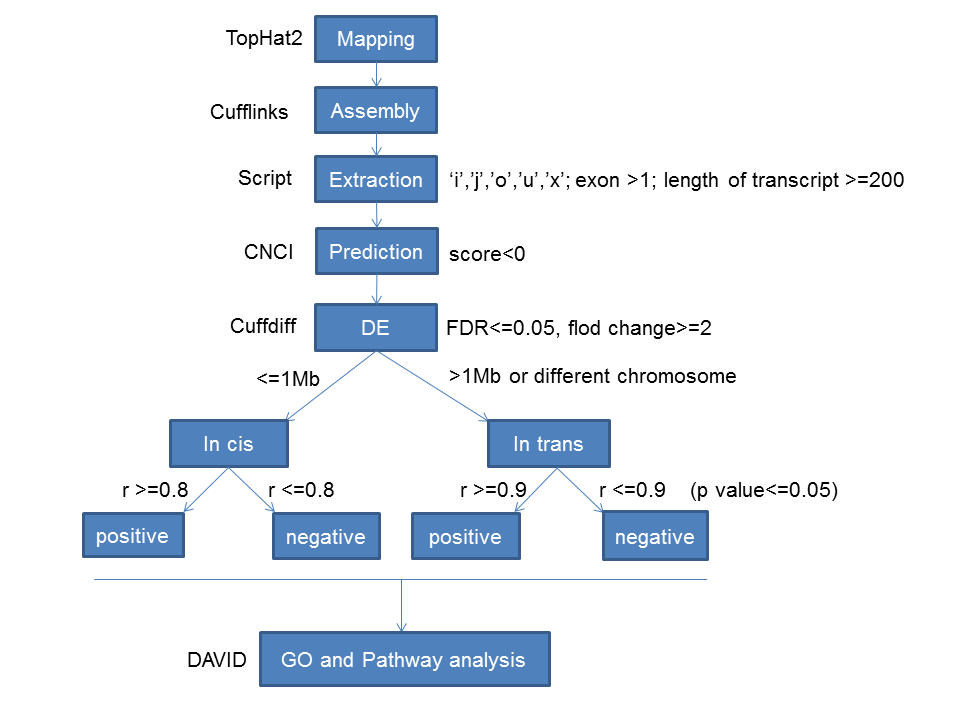


Figure S1. Computational prediction of lncRNAs in HSV-1 infected HFF cells

The computational strategy of predicting of function of lncRNAs in HSV-1 infected HFF cells.


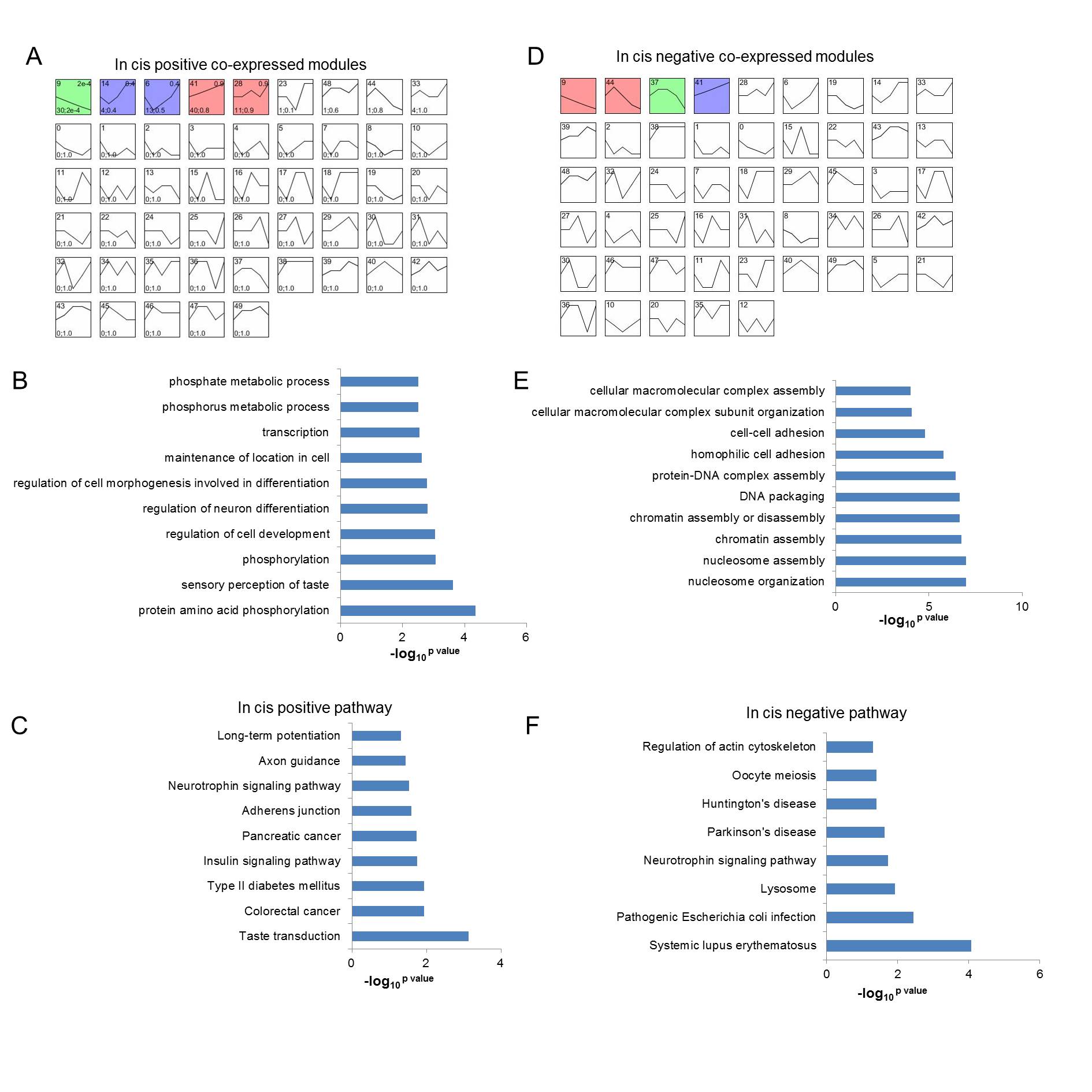


Figure S2. The predicted function of lncRNAs based on co-expressed modules in cis

a. there were 5 significant co-expressed modules in positive regulatory model, p value≤0.05. b-c. GO analysis and pathway analysis of PCGss co-expressed with lncRNAs, p value≤0.05; d. there were 4 significant co-expressed modules in negative regulatory model, p value≤0.05. e-f. GO analysis and pathway analysis of PCGs co-expressed with lncRNAs, p value≤0.05
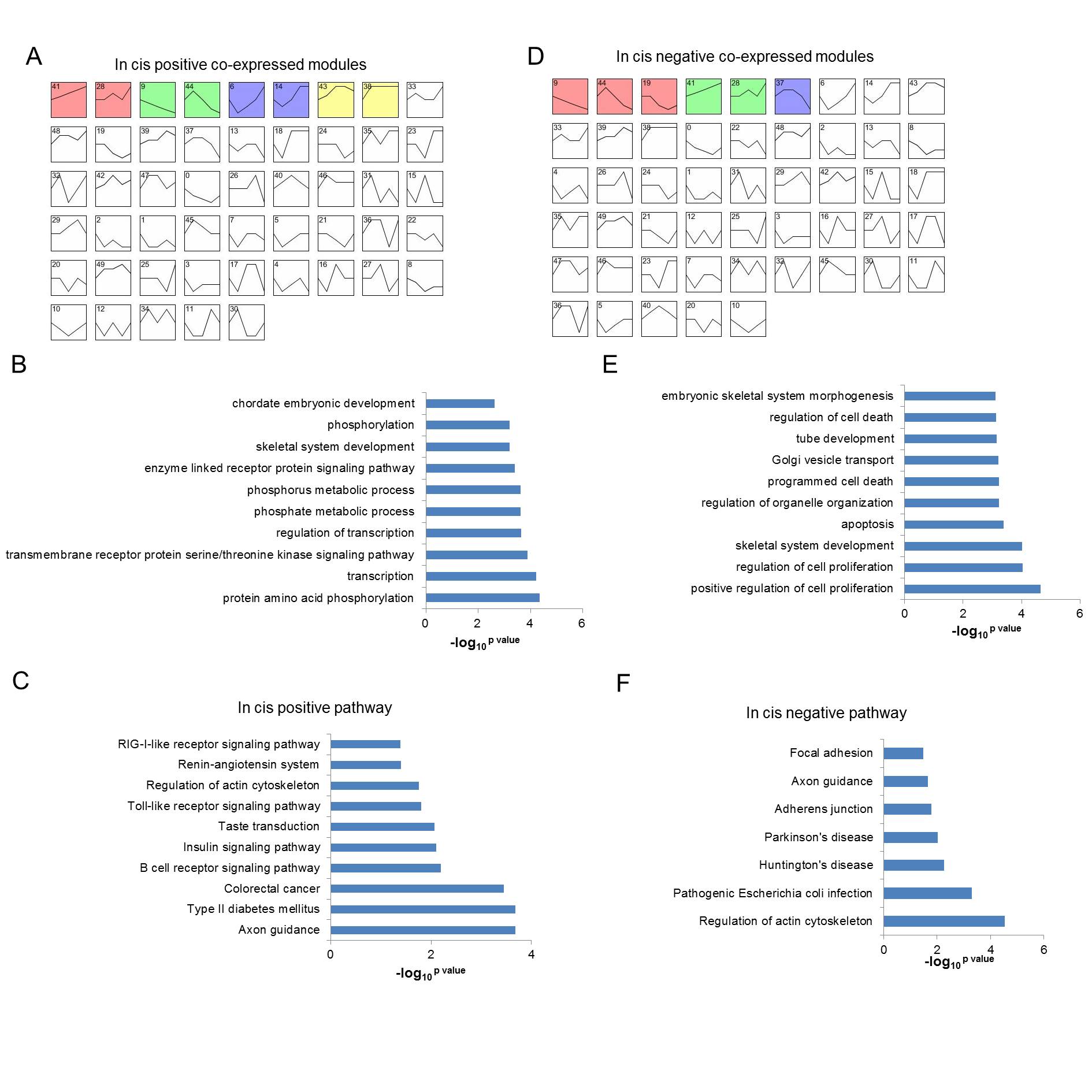


Figure S3. The predicted function of lncRNAs based on co-expressed modules in trans

a. there were 8 significant co-expressed modules in positive regulatory model, p value≤0.05. b-c. GO analysis and pathway analysis of PCGss co-expressed with lncRNAs, p value≤0.05; d. there were 6 significant co-expressed modules in negative regulatory model, p value≤0.05. e-f. GO analysis and pathway analysis of PCGs co-expressed with lncRNAs, p value≤0.05
